# Genetic evaluation and germplasm identification analysis on ITS2, *trn*L-F, and *psb*A-*trn*H of alfalfa varieties germplasm resources

**DOI:** 10.1515/biol-2022-0582

**Published:** 2023-03-21

**Authors:** Wang Yong, Xu Chunbo, Tong Laga, Zhang Xiaoming, Wang Mingjiu

**Affiliations:** College of Grassland, Resources and Environment, Inner Mongolia Agricultural University, Inner Mongolia Autonomous Region, Hohhot, 010011, China; Research Center of Grass Germplasm and Genetic Breeding, Institute of Grassland Research of CAAS, Inner Mongolia Autonomous Region, Hohhot, 010019, China

**Keywords:** alfalfa varieties, ITS2, *trn*L-F, *psb*A-*trn*H, genetic diversity, group identification

## Abstract

In this study, genetic diversity and germplasm identification of 28 alfalfa germplasm cultivars materials were evaluated by analyzing their internal transcribed spacer 2 (ITS2), *trn*L-F, and *psb*A-*trn*H sequences to provide the innovative reference of alfalfa varieties genetic diversity and identify research. The results showed that the fragment average length of ITS2, *trn*L-F, and *psb*A-*trn*H sorting sequences were 455.7 bp, 230.3 bp, and 345.6 bp, respectively. The ITS2 sequence was too conservative to reflect the individual differences between intercultivars and intracultivars in the preliminary experiment. Furthermore, *trn*L-F and *psb*A-*trn*H sequence differences were relatively small between intercultivars but significant between intracultivars. Alfalfa cultivars were divided into four groups by sequence similarity clustering. Alfalfa cultivars *trn*L-F and *psb*A-*trn*H sequences have apparent differences, showing that chloroplast conservative sequences were independent evolution. Compared with *trn*L-F and *psb*A-*trn*H sequences of alfalfa cultivars, *psb*A-*trn*H sequence has abundant variation sites and can better reflect the differences between cultivars than the *trn*L-F sequence. Therefore, the *psb*A-*trn*H sequence can identify different alfalfa cultivars and establish the DNA sequence fingerprint.

## 1 Introduction

Alfalfa (*Medicago sativa* L.) is originated in Asia Minor, the Caucasus, Iran, Turkmenistan, and Central Asia [1] and is one of the most important leguminous forage in the world due to its high yield, high quality, and strong adaptability. *M. sativa* L. is the main research object of breeding, and the resources of alfalfa varieties are the most important basic production materials for animal husbandry and ecological improvement. By 2020, 113 national-level alfalfa varieties have been approved and registered in China [2]. Alfalfa and hybrid alfalfa are the main varieties formed by breeding. Compared with the developed countries in animal husbandry, such as the United States, Canada, New Zealand, and Australia, the number of varieties formed is small, and the breeding efficiency is low. Genetic research and identification of alfalfa germplasm resources have important theoretical and practical significance for alfalfa breeding and the development of the alfalfa industry.

Genetic diversity is the basis of species diversity and ecosystem diversity. It studies the degree of difference between species at the molecular level and reflects the genetic variation within species and between different populations [3]. Research on alfalfa genetics and genetic diversity in China mainly focuses on population morphology, agronomic traits, resistance and physiological characteristics, molecular markers, and DNA sequence analysis. Genetic diversity and identification of cultivar groups based on conserved sequences, i.e., chloroplast and ribosomal genome sequences, have been reported in alfalfa. The chloroplast genome is a closed double-stranded circular molecular structure, generally 115–210 Kb [4]. Chloroplast genes have relatively conservative characteristics, a small genome, a large amount of DNA information, a large copy number, and a high mutation rate of noncoding regions. The *psb*A-*trn*H gene sequence is the gene sequence of the noncoding region of chloroplast RNA, and the noncoding region is the region that cannot be transcribed, which is messenger RNA. The sequence is easy to be amplified and sequenced. It contains a large amount of information and has stable maternal inheritance, which can be efficiently applied to compare differences between different groups within a species. The *trn*L-*trn*F (*trn*L-F) gene sequence is the chloroplast RNA coding gene and the noncoding region gene sequence [5]. Sequence fragments have the advantages of simple sequence, convenient amplification, high evolution rate, little influence on the external environment, and easy mutation and variation of bases between different populations. It is widely used in interspecific and subspecific level genetic evolution phylogeny research. The chloroplast gene *trn*L-F sequence, *psb*A-*trn*H sequence, and nuclear gene internal transcribed spacer (ITS) sequence marker systems are mainly used in plant phylogeny research, species genetic diversity, and medicinal use in China. There are few reports on identifying plant Chinese medicinal materials in the herbage field. For example, datureae plants analysis, *Bupleurum marginatum* var. analysis based on internal transcribed spacer 2 (ITS2) barcode [6,7], mainly concentrates on forage and the phylogenetic research of Poaceae materials. In relevant studies, phylogenetic analysis has been conducted on the materials of the genus *Alkali*, the genera *Astragali radix*, the *Medicago*, and their relatives [8,9,10,11,12,13]. Ribosomal DNA ITS sequences are a family of genes encoding ribosomal RNA in the nucleus of plant cells and are nuclear gene fragments [6]. The sequence of the ribosome coding region is generally highly conserved, and there are few reports on the study of the conserved ribosome sequence in alfalfa in China. In this study, 28 alfalfa varieties and germplasm materials were taken as the research objects, and their ITS2, *trn*L-F, and *psb*A-*trn*H sequences were analyzed for genetic and germplasm identification. The genetic structure characteristics and genetic diversity of alfalfa variety germplasm resources in different populations were analyzed. The research has a theoretical reference for screening excellent alfalfa germplasm, establishing an alfalfa variety identification system, and exploring and utilizing alfalfa variety germplasm resources. Furthermore, it fills the gap of research on the genetic and variety identification characteristics of alfalfa in China combined with *trn*L-F, *psb*A-*trn*H, and other conserved sequences.

## 2 Materials and methods

### 2.1 Experiment material

The test materials are 28 alfalfa varieties (materials) from different countries and regions (Table 1). The seeds of the experimental germplasm resources were obtained from the National Medium-term Forage Germplasm Resource Bank of the Grassland Research Institute, Chinese Academy of Agricultural Sciences. The test material was planted in the Agricultural and Animal Husbandry Interlaced Area Experimental Demonstration Base of the Grassland Research Institute of the Chinese Academy of Agricultural Sciences in 2012 and was planted as a single plant. In 2018, alfalfa was sampled at the branching stage, and 10 fresh and young leaf samples were collected from each material, placed in an ice box, and brought back. Then, samples were stored in a −80°C ultra-low temperature refrigerator in the laboratory for future use.

**Table 1:** Alfalfa varieties (materials)

Code	Name	Species	Origin	Analysis method
1	Aohan	*Medicago sativa* L.	China	ITS2
2	Junggar	*Medicago sativa* L.	China	*trn*L-F, *psb*A-*trn*H
3	Zhaodong	*Medicago sativa* L.	China	*trn*L-F, *psb*A-*trn*H
4	Tianshui	*Medicago sativa* L.	China	*trn*L-F
5	Longdong	*Medicago sativa* L.	China	*psb*A-*trn*H
6	Xinjiang Daye	*Medicago sativa* L.	China	*trn*L-F, *psb*A-*trn*H
7	Gannong No. 3	*Medicago sativa* L.	China	*trn*L-F, *psb*A-*trn*H
8	Zhongmu No. 2	*Medicago sativa* L.	China	*trn*L-F, *psb*A-*trn*H
9	Tumu No. 1	*Medicago varia* Martin.	China	*trn*L-F, *psb*A-*trn*H
10	Xinmu No. 1	*Medicago varia* Martin.	China	*trn*L-F, *psb*A-*trn*H
11	Caoyuan No. 1	*Medicago varia* Martin.	China	*trn*L-F, *psb*A-*trn*H
12	Caoyuan No. 2	*Medicago varia* Martin.	China	ITS2, *trn*L-F, *psb*A-*trn*H
13	Gannong No. 1	*Medicago varia* Martin.	China	*trn*L-F, *psb*A-*trn*H
14	Gannong No. 2	*Medicago varia* Martin.	China	*trn*L-F, *psb*A-*trn*H
15	Japan 90	*Medicago sativa* L.	Japan	*trn*L-F
16	Bear No. 1	*Medicago sativa* L.	US	*psb*A-*trn*H
17	Czech 26-1	*Medicago sativa* L.	Czech	*trn*L-F, *psb*A-*trn*H
18	Golden Queen	*Medicago sativa* L.	US	*psb*A-*trn*H
19	Soviet Union 1209	*Medicago sativa* L.	Russia	*trn*L-F, *psb*A-*trn*H
20	Soviet Union 6220	*Medicago sativa* L.	Russia	*trn*L-F, *psb*A-*trn*H
21	UK	*Medicago sativa* L.	UK	*psb*A-*trn*H
22	AmeriGraze 401Z	*Medicago sativa* L.	US	*trn*L-F
23	Apollo supreme	*Medicago sativa* L.	US	*trn*L-F, *psb*A-*trn*H
24	Rumble	*Medicago sativa* L.	Canada	*trn*L-F, *psb*A-*trn*H
25	WL-320	*Medicago sativa* L.	US	*trn*L-F
26	WL-323	*Medicago sativa* L.	US	*trn*L-F, *psb*A-*trn*H
27	Canada	*Medicago sativa* L.	Canada	*trn*L-F, *psb*A-*trn*H
28	*Medicago falcata*	*Medicago falcata* L.	China	*trn*L-F, *psb*A-*trn*H

**Figure 1:**
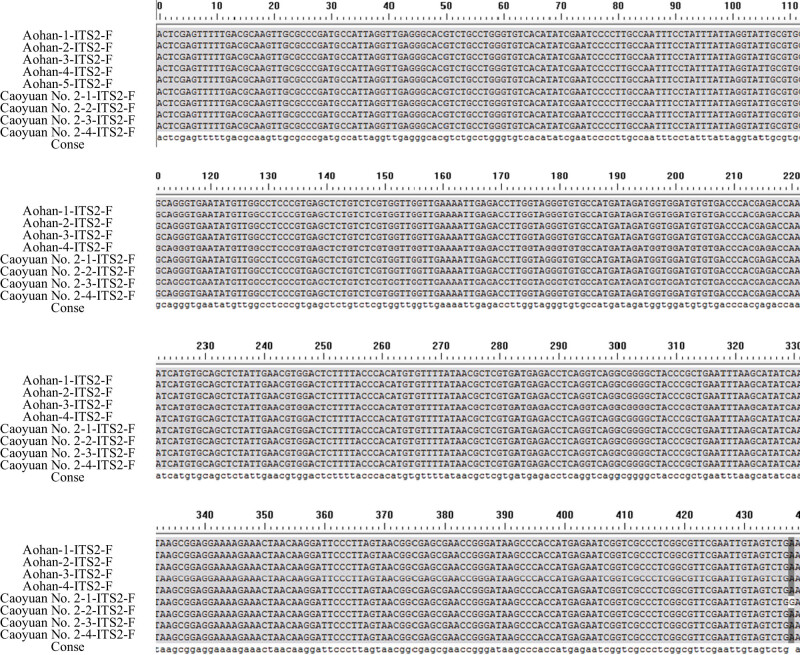
ITS2 sequences of different alfalfa varieties (materials).

There were differences in the number of materials selected for the alfalfa varieties tested by different sequences, and two materials were selected for ITS2 (Table 1). Among them, the ITS2 sequence research was conducted on *Medicago sativa* L.cv.Aohan and *Medicago varia* Martin.cv.Caoyuan No. 2, and Aohan and Caoyuan No. 2 are two species, and there should be big differences in gene sequences, but two copies were found in the preliminary identification test. The DNA sequences of the materials showed little difference (Figure 1). Therefore, other corresponding material experiments were not carried out in the follow-up. The ITS2 sequence was unsuitable for analyzing genetic diversity among alfalfa varieties or species. According to the presence or absence of materials in the research process, 24 alfalfa materials were selected for the *psb*A-*trn*H sequence, and 23 materials were selected for the *trn*L-F sequence.

### 2.2 DNA extraction

The DNA extraction method of single leaf material was extracted by DNA extraction kit (CTAB Plant genome extraction kit, BLKW, Beijing). DNA concentration and purity were detected by the 1.0% agarose detection method and trace UV/Vis spectrophotometer, respectively.

### 2.3 Primer and polymerase chain reaction (PCR) amplification procedure

The primers used in the experiment are shown in Table 2. The research results of NCBI number are presented in Table 2 (list first number only). ITS2 sequence PCR amplification system 30 μL includes 10–20 ng template 1 μL, 10 mol L^−1^ primers 1 μL each, PCR buffer 3 μL, dNTP 1 μL, Mg^2+^ 2 μL, Taq DNase 1 μL, and ddH_2_O 20 μL. The amplification program was an annealing temperature of 56°C, pre-denaturation at 94°C for 5 min, denaturation at 94°C for 30 s, annealing at 56°C for 30 s, extension at 72°C for 1.5 min, 40 cycles, and extension at 72°C for 10 min. *trn*L-F, *psb*A-*trn*H gene interval sequence amplification system 30 μL includes 10–20 ng template 1.5 μL, PCR Mix 15 μL, ddH_2_O 10.5 μL, primer F 1.5 μL, and primer R 1.5 μL. Program: pre-denaturation at 94°C for 3 min, denaturation at 94°C for 30 s, annealing at 49°C for 30 s, extension at 72°C for 90 s, 30 cycles, and extension at 72°C for 7 min. All PCR amplification products were stored in a refrigerator at 4°C for future use.

**Table 2:** Primer message

Sequence	Primer (5′ to 3′)	Temperature (°C)	Amplicon size (bp)
ITS2, NCBI:MT610943.1…	F: ATGCGATACTTGGTGTGAAT	56	20
	R: GACGCTTCTCCAGACTACAAT		21
*trn*L-F, NCBI:MW271002.1…	F: GTTATGCATGAACGTAATGCTC	49	22
	R: CGCGCATGGTGGATTCACAATCC		23
*psb*A-*trn*H, NCBI:KP174827.1…	F: GGTTCAAGTCCCTCTATCCC	49	20
	R: ATTTGAACTGGTGACACGAG		21

*trn*L-F/*psb*A-*trn*H sequence PCR 30 μL reaction system includes 1.5 μL diluted DNA solution, 15 μL PCR Mix, 10.5 μL ddH_2_O, 1.5 μL primer F, and 1.5 μL primer R. They were mixed to form the reaction system and centrifuged for use. The amplification program was optimized for reaction conditions. The amplification is performed as follows: pre-denaturation at 94°C for 3 min, denaturation at 94°C for 30 s, annealing at 49°C for 30 s, and extension at 72°C for 30 cycles for 90 s, and then extended at 72°C for 7 min and stored at 4°C for future use.

The amplified products were subjected to 1.0% agarose gel electrophoresis, stained with nucleic acid dyes, and observed and photographed with a gel imaging system.

### 2.4 PCR product sequencing

The PCR amplification products were purified and used for the sequencing reaction. The ITS2, *trn*L-F, and *psb*A-*trn*H sequences of all product samples were determined by Shenzhen Huada Gene Technology Co., Ltd. Sequencing was performed by direct sequencing of PCR products, and each sample was sequenced in forward and reverse directions to ensure the sequencing accuracy.

### 2.5 Data analysis

The DNA sequence fragments obtained by sequencing were used for sequencing quality evaluation. Forward and reverse sequence sequencing results were analyzed by Codoncode Aligner, seqMan, and DNAMan. Furthermore, CLUSTALX 2.0 software was used for sequence alignment. The genetic distances of the aligned sequences were calculated by MEGA 7.0 software, and the molecular phylogenetic tree was established by the kimura method. The confidence of each branch of the phylogenetic tree was tested by bootstrap (1,000 repetitions), and gaps were always treated as missing.

## 3 Results and analysis

### 3.1 ITS2 sequence polymorphism analysis of alfalfa

Aohan and Caoyuan No. 2 were selected as research materials, and 18 forward and reverse ITS2 sequences were obtained by PCR amplification, recovery, and sequencing. The sequencing results of the nine genotypes ITS2 with unidirectional sequences are shown in Figure 1. The results showed that the effective sequence was 454–457 bp in full length, with an average of 455.7 bp. The nucleic acid bases of the two alfalfa populations differed very little. The contents of T, C, A, and G were 26.0, 22.3, 24.2, and 27.5%, with variations ranging from 25.9∼26.2, 22.1∼22.7, 23.8∼24.5, and 27.2∼27.9%, respectively. The average content of A and T is 50.2%, which is slightly larger than that of G and C. It can be seen from the unidirectional sequence of ITS2 that the corresponding sequences of Aohan and Caoyan No. 2 have basically no sequence differences between populations and local populations after removing the primer sequences and irregular sequences at the initial stage of sequencing. This result indicated that ITS2 was too conservative for alfalfa resources. In addition, no insertion and deletion variation was found between the corresponding sequences, and the corresponding ITS2 sequences were not suitable for application as genetic diversity markers. Aohan and Caoyuan No. 2 are *M. sativa* L. and *Medicago varia* Martin, which are different alfalfa species. The ITS2 sequences show identical sequences among species, i.e., no difference between species. In the selected five Aohan and four Caoyuan No. 2, the intraspecific sequences are also the same, i.e., no intraspecific difference. There is no polymorphism in applying the ITS2 sequence in alfalfa to study interspecific and intraspecific genetic diversity.

### 3.2 *trn*L-F sequence polymorphism analysis of alfalfa

**Figure 2:**
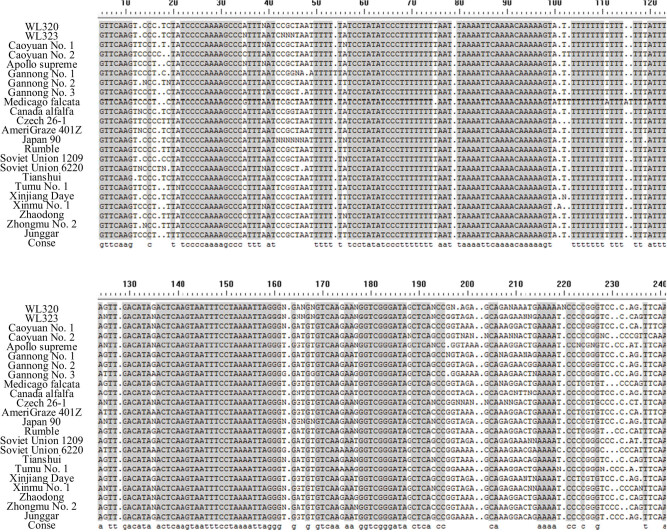
*Trn*L-F sequences of different alfalfa varieties (materials).

A total of 115 forward and reverse *trn*L-F sequences were obtained by PCR amplification, recovery, and sequencing of 23 alfalfa germplasm materials. After removing primer sequences, hybrid and repetitive sequence fragments, 23 alfalfa materials (genotypes), and *trn*L-F unidirectional sequences were sorted out. The results are shown in Figure 2. The effective sequence length is about 240 bp, averaging 230.3 bp. Nucleic acid bases of alfalfa materials are different. The average content of G + C is 32.4%, and the variation is within 30.6–34.6%. The average ambiguous site rate in the spliced sequences is 1.59%. The polymorphism analysis of *trn*L-F sequences of 23 alfalfa germplasm materials showed that the total number of sequence loci is 242, the haplotype diversity (Hd) is 0.478, and the nucleotide polymorphism (Pi) is 0.322, with an average number of nucleotide differences (k) of 1.974.

### 3.3 *psb*A-*trn*H sequence polymorphism analysis of alfalfa

**Figure 3:**
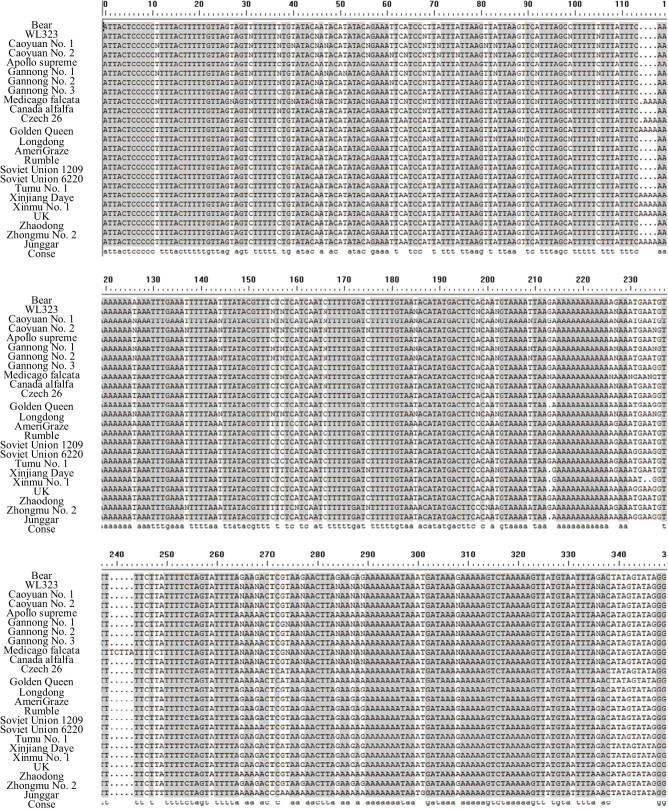
*Psb*A-*trn*H sequences of different alfalfa varieties (materials).

A total of 120 forward and reverse *psb*A-*trn*H sequences were obtained by PCR amplification, recovery, and sequencing of *psb*A-*trn*H sequences of 24 alfalfa germplasm materials. After removing primer sequences, hybrid and repetitive fragments, 24 unidirectional sequences of *psb*A-*trn*H sequences of materials (genotypes) were sorted out. The results are shown in Figure 3. The effective sequence length is about 345 bp, with an average of 345.6 bp. The average content of G + C in the alfalfa *psb*A-*trn*H sequence is 18.27%, and the variation is within 21.4–17.6%. The average ambiguous site rate in the spliced sequences is 8.96%. The polymorphism analysis of *psb*A-*trn*H sequences of 24 alfalfa germplasm materials showed that the total number of loci is 352 bp, the haplotype diversity (Hd) is 0.582, and the nucleotide polymorphism (Pi) is 0.457, with an average number of nucleotide differences (k) of 2.682. The total number of variation sites (Vs) in the *psb*A-*trn*H sequence of each material is 86, accounting for 25.13% of the total number of sites. There are 44 single nucleotide variation sites (Ss) and 13 parsimony informative sites (Ps). Among the 84 single-nucleotide variation sites, 17 are two-base variations. The type of sequence base variation is mainly substitution variation, including T-C, G-A transition, T-A, G-T, C-A transversion, and 2–4 bp insertion and deletion variation.

### 3.4 Cluster analysis of *trn*L-F and *psb*A-*trn*H sequence in alfalfa

**Figure 4:**
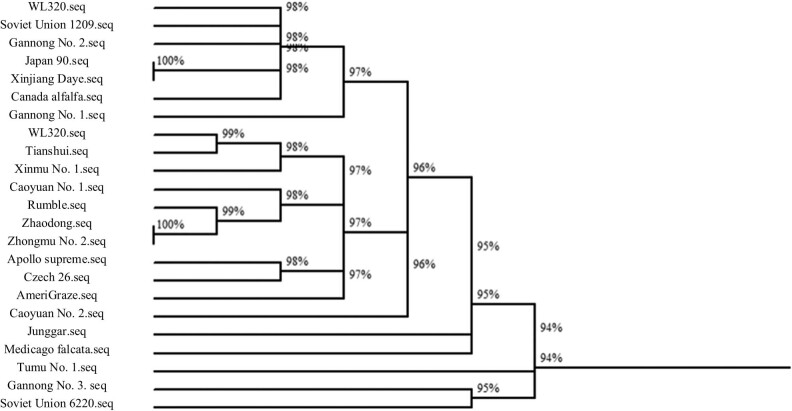
Alfalfa similarity clustering of *trn*L-F sequences.

The similarity of *trn*L-F sequences of 23 alfalfa germplasms was clustered (Figure 4). Analysis showed that the *trn*L-F sequence homology similarity between Japan 90 and Xinjiang Daye as well as Zhaodong and Zhongmu No. 2 is the largest, both of which are 100%. Tumu No. 1 and other alfalfa varieties have the lowest sequence homology similarity of *trn*L-F, with a similarity of 94%. The *trn*L-F sequence homology of *M. falcata* and most materials is low, ranging from 94 to 95%.

The test materials were clustered using the *trn*L-F sequence homology similarity of 96% as the classification standard. Among them, the materials (varieties) such as Junggar, *M. falcata*, Tumu No. 1, Gannong No. 3, and Soviet Union 6220 were grouped into one category, and other varieties were grouped into one category.

The clustering results of *trn*L-F sequence homology similarity between *M. falcata* and other test materials did not reach the outgroup (homogeneity) level with the highest sequence similarity. This result reflects that the *trn*L-F sequence is relatively conserved in alfalfa species such as *M. falcata* L. and *M. sativa* L. Comparison of *M. falcata trn*L-F sequence with other *trn*L-F sequences showed that ATTT at the 100 bp site and AT at the 116–117 bp site are specific bases and can be used as the basis for the identification of *M. falcata* L. and *M. sativa* L. resources.

**Figure 5:**
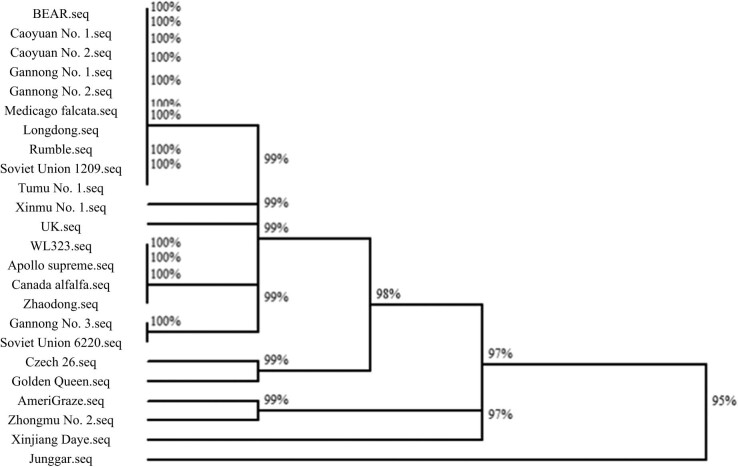
Alfalfa similarity clustering of *psb*A-*trn*H sequences.

Figure 5 shows that the similarity of *psb*A-*trn*H sequences of 24 alfalfa germplasms was clustered. Analysis suggested that the homology among the alfalfa varieties Bear No. 1, Caoyuan No. 1, Caoyuan No. 2, Gannong No. 1, Gannong No. 2, *M. falcata*, Longdong, Rumble, Soviet Union 1209, Tumu No. 1, and WL-323, Apollo supreme, Canadian alfalfa and Zhaodong alfalfa, Gannong No. 3, and Soviet Union 6220 alfalfa groups are the highest, all of which are 100%. The *psb*A-*trn*H sequence homology similarity between Junggar alfalfa and other alfalfa is 95%. With the *psb*A-*trn*H sequence homology similarity of 96% as the classification criterion, the test materials can be grouped into one category except for Junggar alfalfa. The *psb*A-*trn*H sequence homology of alfalfa and most alfalfa materials can reach 100%. The average length of the *psb*A-*trn*H sequence is 345.6 bp. The sequence is rich in polymorphisms such as single-nucleotide variation sites, parsimony information sites, and insertion and deletion fragments. The *psb*A-*trn*H sequencing results can better identify alfalfa variety resources and can be applied to alfalfa variety identification.

### 3.5 Sequence comparison of *trn*L-F and *psb*A-*trn*H in alfalfa and DNA barcode formation analysis

The *trn*L-F sequences of 24 alfalfa varieties (materials) and the *psb*A-*trn*H sequences of 23 alfalfa varieties (materials) show sequence differences. The corresponding sequences can form DNA sequence barcodes to identify alfalfa varieties. According to Figures 2 and 3, the *psb*A-*trn*H sequence variation sites are abundant for all sequenced alfalfa materials. The *trn*L-F sequence forms 67 variation sites and 84 *psb*A-*trn*H sequences, according to statistics. Table 3 shows that the sequencing fragments are sorted and aligned within a single variety, and the *psb*A-*trn*H variation sites of the materials within varieties range from 0 to 5, with an average of 0.79. The *trn*L-F variant sites range from 0 to 14, averaging 5.09. Among the variation rates of *trn*L-F and *psb*A-*trn*H sequences within varieties, the average content of G + C fragments of *trn*L-F sequences in each variety is 32.4%. The highest single nucleotide variation rates among varieties are shown in Zhongmu No. 2 and Canadian alfalfa, both with 1.46%. The average G + C content of the *psb*A-*trn*H sequence fragment is 18.3%. The highest intravariety variation rate is Czech 26-1 alfalfa, with a variation rate of 4.78%. Based on the comparison results of alfalfa DNA sequences between and within varieties, the DNA identification barcodes of alfalfa *psb*A-*trn*H sequences are more abundant in polymorphisms between varieties. In addition, the variation rate within varieties is relatively low, and the identification of alfalfa varieties has formed a clearer DNA barcode sequence.

**Table 3:** Basic information of alfalfa varieties *psb*A-*trn*H and *trn*L-F sequences

Name	*psb*A-*trn*H				*trn*L-F			
	Fragment	G + C	Mutation	Mutation rate	Fragment	G + C	Mutation	Mutation rate
Golden Queen	341	16.72	0	0	—	—	—	—
Caoyuan No. 1	341	17.01	0	0	233	32.62	10	4.29
Caoyuan No. 2	341	17.01	0	0	233	32.62	10	4.29
Apollo supreme	341	19.06	0	0	232	30.6	8	3.45
Gannong No. 1	341	17.01	1	0.29	231	32.47	4	1.73
Gannong No. 2	341	17.01	2	0.59	231	33.33	2	0.87
Gannong No. 3	341	19.06	0	0	230	33.04	2	0.87
*Medicago falcata*	349	16.91	5	1.43	231	33.33	1	0.43
Canada alfalfa	341	17.6	5	1.46	230	33.04	5	2.17
Czech 26-1	345	18.26	0	0	230	31.74	11	4.78
Rumble	341	21.11	0	0	230	32.17	1	0.43
Soviet Union 1209	341	21.41	0	0	229	33.19	3	1.31
Soviet Union 6220	341	19.35	0	0	230	33.04	3	1.3
Tumu No. 1	341	21.41	0	0	231	31.6	4	1.73
Xinjiang Daye	345	19.71	2	0.58	229	32.31	5	2.18
Xinmu No. 1	343	20.99	0	0	231	32.03	9	3.9
Zhaodong	341	18.77	0	0	229	34.06	3	1.31
Zhongmu No. 2	341	20.53	4	1.46	230	32.17	4	1.74
Junggar	345	18.84	0	0	233	33.05	0	0
AmeriGraze	341	21.41	0	0	231	32.03	1	0.43
BEAR	342	21.05	0	0	—	—	—	—
WL323	341	17.3	0	0	232	32.76	4	1.72
Longdong	341	19.94	0	0	—	—	—	—
UK	344	21.22	0	0	—	—	—	—
WL320	—	—	—	—	222	32.88	9	4.05
Japan 90	—	—	—	—	228	30.7	14	6.14
Tianshui	—	—	—	—	229	32.31	4	1.75

## 4 Discussion

With the innovation and progress of identification technology, fingerprints and DNA barcoding have become the core research fields of identification technology. They are widely used in biological research and are considered the most efficient and accurate technical system for identifying biogenetic characteristics from root genes and biological functional tissues. The quality of alfalfa cultivar resources determines the yield potential and resistance to a large extent. Research on the development of an accurate, reliable, rapid, and simple variety identification method is important for identifying the authenticity of alfalfa varieties, strengthening the construction of the seed quality standard system, and improving the quality of alfalfa seeds. Currently, the identification of alfalfa varieties mainly relies on conventional identification methods. The corresponding fingerprint technology is affected by factors such as the detection object, technology, and equipment, and few standardized detection procedures have been established. Standardized test procedures need to be strengthened. Fingerprints and DNA barcoding have the advantages of rapidity, accuracy, and little impact on environmental pressures in identifying the genetic variation and genetic composition of alfalfa varieties.

Although many scientists have done a lot of research on DNA fingerprints, the identification of alfalfa varieties is still a difficult problem. This article mainly explored the use of ITS2, *trn*L-F, and *psb*A-*trn*H markers to identify alfalfa varieties. We use only 28 genotypes, but the technology we have established can group these alfalfa varieties well. The preliminary verification of these technologies shows that this is of great value for the identification of alfalfa varieties and is worth promoting and exploring.

Ribosomal DNA ITS is a family of genes encoding ribosomal RNA in the nucleus of plant cells [14]. Ribosomal coding region sequences are generally highly conserved, similar to chloroplast-conserved sequences. The corresponding spacer sequence is characterized by a large amount of mutation information, rapid mutation, simple sequences, convenient amplification, high evolution rates, less influence by the external environment, and no functional restrictions. Currently, it is widely used in the study of genetic evolutionary phylogeny at the interspecific and subspecific levels. In reference [15], six Leguminosae forages were tested, and ITS cannot be used as DNA barcoding candidate sequences due to low amplification efficiency. In reference [16], 156 alfalfa populations were tested. The ITS fragments of other species, except for a few relatives, also showed a high ability of species to define. The results of the former are preferred. Comparing the results of different studies revealed that the selected primer fragments differ, showing different results. The screening and identification of primers are critical. Comprehensive analysis showed that the ITS2 primer used in this research needs to be further optimized, and screening should be performed in many species, i.e., to distinguish the DNA barcoding potential of different alfalfa varieties.

Chloroplast gene *trnL-F* and *psbA-trnH* sequences are chloroplast spacer gene fragments with high amplification success rate and short purpose fragment length [17]. The chloroplast gene *trn*L-F and *psb*A-*trn*H sequence and nuclear gene ITS sequence marker systems are mainly used in plant phylogeny research, species genetic diversity, and identification of medicinal plants in china. Among them, the systematic classification of medicinal plants and the identification of Chinese medicinal materials are the most widely used, and the corresponding technology tends to be mature. Currently, Rosaceae, Rutaceae, Honeysuckle, Ginseng, Cistanche, Euphorbia, Shegan, Chonglou, and other families, genera, and species have initially established DNA barcodes and fingerprints based on conserved chloroplast sequences [18,19,20,21,22,23,24]. The genetic diversity and molecular phylogenetic classification of medicinal plant germplasm resources are mainly carried out in the families, genera, and species of sage, *Schisandra*, *Trillium*, *Ophiopogon japonicus*, Crow garlic, tulip, and onion [25,26,27,28]. The chloroplast gene *trn*L-F, *psb*A-*trn*H sequence, and the nuclear gene ITS sequence marker systems are rarely reported in forages in China, mainly in Sichuan Agricultural University and Lanzhou University, with more phylogenetic studies in *gramineae* materials. In reference [7], ITS sequences and *psb*A-*trn*H sequences were used for phylogenetic analysis on 37 materials of 6 genera, including Elymus. In reference [8], the *psb*A-*trn*H sequence was used to study the genetic diversity of 87 populations of *M. japonica*. In reference [9], *ndh*F, *psb*A-*trn*H, and *trn*L-F gene sequences were adopted to study the developmental phylogeny of 67 materials of the genus *Gossypium* and its relatives. In reference [6], ITS sequences were used to conduct phylogenetic analysis on 41 materials of the genus *Laysporium* and its relatives. The application of legume and alfalfa mainly includes the classification of pathogen populations, the legume phylogenetic relationships, and species delimitation [29,30,31]. In reference [16], samples representing 21 naturally distributed species in China were collected, and the chloroplast genomes of 75 individuals representing 20 species were assembled. The results showed that 18 species are well delimited except for *Medicago sativa*, *Medicago falcata*, and *Melilotus officinalis*. The primers used in this study verified the feasibility of identifying alfalfa species as DNA barcoding and formed alfalfa variety identification barcoding using *psb*A-*trn*H and *trn*L-F. Compared with the 23 materials used in the study, *psb*A-*trn*H sequence identification was clearer and more accurate, and the effect of distinguishing different alfalfa varieties was better. The studies on genetic diversity and variety identification of alfalfa have a one-sided analysis regarding morphological characteristics, agronomic traits, functional proteins, isozymes, and molecular markers. Conservative gene sequences can effectively complement their research shortcomings. To study the new and effective methods of alfalfa genetic diversity, further in-depth related research can be conducted to make the research methods and technical means more suitable for the research of alfalfa genetic diversity and variety identification.

## 5 Conclusion

In this study, the widely used sequences of ribosomal ITS2 and chloroplast *trn*L-F and *psb*A-*trn*H were used to analyze the genetic diversity and variety identification of alfalfa varieties. The alfalfa ITS2, *trn*L-F, and *psb*A-*trn*H sequences were sequenced to obtain the sequenced fragments with average lengths of 455.7 bp, 230.3 bp, and 345.6 bp, respectively. Among them, the alternative fragments of the ITS2 sequence showed high conservation both within and among varieties. The *trn*L-F and *psb*A-*trn*H sequences differed little within varieties but had obvious differences among varieties. According to the alfalfa similarity clustering of *trn*L-F and *psb*A-*trn*H sequences, the tested alfalfa varieties were clustered into four categories. The similarities of *trn*L-F and *psb*A-*trn*H sequences were different among alfalfa varieties, and the differences were large, indicating that the conserved chloroplast sequences in each variety population were independently evolved. The comparison of the *trn*L-F and *psb*A-*trn*H sequences of alfalfa varieties showed that the *psb*A-*trn*H sequence had more variant sites and richer sequence polymorphisms. Thus, the sequence is more suitable for forming clear DNA barcoding for alfalfa variety identification. During alfalfa variety identification and genetic diversity analysis, the ITS2 sequence was too conservative. The initial sequencing results in the previous experiment could not reflect the differences between varieties and individuals within varieties. The corresponding polymorphisms of the chloroplast gene sequences are abundant, which can better reflect the differences between varieties and are more suitable for identifying different alfalfa varieties.
